# Non-contact robotic manipulation of floating objects: exploiting emergent limit cycles

**DOI:** 10.3389/frobt.2023.1267019

**Published:** 2023-10-12

**Authors:** Sylvain Jacquart, Nana Obayashi, Josie Hughes

**Affiliations:** CREATE Lab, Institute of Mechanical Engineering, EPFL, Lausanne, Switzerland

**Keywords:** limit cycles, water interactions, non-contact manipulation, floating objects, ocean engineering

## Abstract

The study of non-contact manipulation in water, and the ability to robotically control floating objects has gained recent attention due to wide-ranging potential applications, including the analysis of plastic pollution in the oceans and the optimization of procedures in food processing plants. However, modeling floating object movements can be complex, as their trajectories are influenced by various factors such as the object’s shape, size, mass, and the magnitude, frequency, and patterns of water waves. This study proposes an experimental investigation into the emergence ofrobotically controlled limit cycles in the movement of floating objects within a closed environment. The objects’ movements are driven by robot fins, and the experiment plan set up involves the use of up to four fins and variable motor parameters. By combining energy quantification of the system with an open-loop pattern generation, it is possible to demonstrate all main water-object interactions within the enclosed environment. A study using dynamic time warping around floating patterns gives insights on possible further studies.

## 1 Introduction

Many aquatic animals exhibit tool-use-like behavior using water itself as a “tool” for self-protection and hunting (Mann and Patterson, 2013). Octopuses and squids squirt water jets as a means of attack or to propel itself to aid in burrowing for camouflage (Wells, 1990; Mather and Dickel, 2017). Dolphins and whales use waves, bubbles, and mud for hunting (Torres and Read, 2009), one of the most famous of them being the bubble-net feeding technique by humpback whales (Friedlaender et al., 2011). Interacting with objects and surroundings without direct physical contact—or non-contact manipulation—enables these animals to perform tasks that would be otherwise impossible using only their flippers or fins. This is also true for artificial systems which leverage the fluidic environment to manipulate objects. Manipulation of these objects usually occur as a result of transfer of energy through the fluid medium. Oftentimes, as a result of nonlinear interactions within dynamic systems, limit cycles, or recurring patterns of behavior, may arise spontaneously. These can emerge not only in biological rhythms (Asgari-Targhi and Klerman, 2019) or oscillations (Wallace et al., 2011), but also in robotic systems, primarily as a control strategy to achieve stable and repetitive motions (Dai and Tedrake, 2012; Lee et al., 2019). In the context of a controlled water environment, such as a tank, understanding the emergence of limit cycles in the movements of floating objects when the water environment is actuated allows for precise object manipulation by harnessing stable and predictable oscillations, enabling control over object positions and trajectories, without the need for direct contact.

Modeling the movements of floating or deformable objects in the fluid environment is challenging (Persi et al., 2020; Obayashi et al., 2022), as they are influenced by various factors including the object’s morphology and the magnitude, frequency, and patterns of water waves. While mathematical modeling of floating objects on open water bodies is known (Marchenko, 1999; Raman-Nair and Chin, 2012), there exists a significant reality gap when validating through experimentation. Existing non-contact manipulation techniques that rely on levitation in the chemical and pharmaceutical fields (Al-Nuaimi et al., 2022) are limited in their capacity to manipulate a wide range of materials. A number of other approaches work on a microscale, or have a very small range of non contact capabilities (Floyd et al., 2009; Peyer et al., 2011). Manipulation of free-floating objects leveraging Faraday flows and learning techniques require a large amount of training data (Hardman et al., 2022). In order to evaluate the potential usage of non-contact manipulation techniques for applications in fields such as food grading and sorting (Abbas et al., 2019) and plastic waste gathering in bodies of waters^1^, we must be capable of quantifying the behavior and interactions of floating object morphology in the fluid environment. This is a question which is of key relevance to the soft robotics field (Stella and Hughes, 2023).

In this work, we leverage a closed water environment where we induce limit cycles in floating object trajectories by actuating the water’s surface using fins. Our goal is to explore the potential of controlling these object movements without the need for direct contact. The interactions between the floating objects and their surrounding environment is experimentally characterized. Using dynamic time warping (DTW) to compare the trajectories of the floating objects enables quantification of the similarities (Ilic et al., 2023) between their limit cycles, which can then be used to generate an open-loop controller for controlling the objects’ trajectories. In the remainder of the paper, we present the methods and experimental setup used to explore these limit cycles, followed by experimental results and conclusion with suggestions for future work.

## 2 Materials and methods

In this section we first introduce the robotic experimental setup developed to investigate non-contact manipulation and the emergence of limit cycles. The analysis approach is then detailed to explore the controllability of objects in this manner.

### 2.1 Experimental setup, fins and control parameters

**FIGURE 1:**
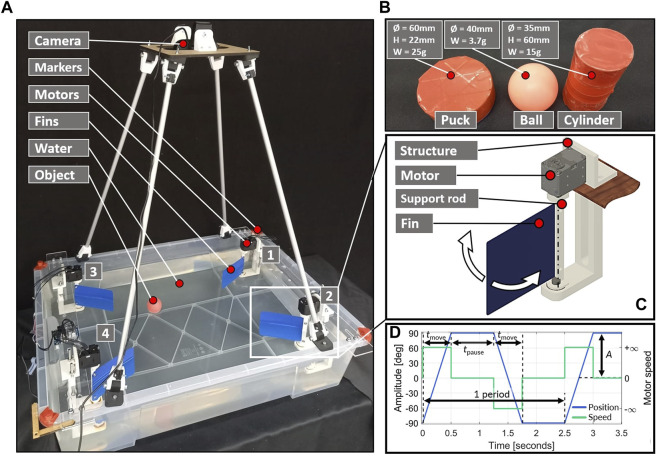
Experiment setup for this study, with the 4 fin setups in the corners **(A)**, selected floating objects with dimensions **(B)**, motor and fin system placed in corners, which allows only for rotation in around the rod axis **(C)**, and parameterized controller plot according to standard motor parameters **(D)**.

In order to observe non-contact manipulation and the emergent limit cycles of floating objects, a custom setup is built as shown in Figure 1A. The rectangular tank is filled with 50 L of water and a camera is placed 70 cm above the water surface. Non-contact interactions are generated using rectangular 0.4 mm-thick polypropylene sheets and are individually actuated by DYNAMIXEL XL430-W250-T around their support rod (Figure 1C). The primary control parameters are the fin amplitude, $A\in \left[20{}^{\circ},\;90{}^{\circ}\right]$ , the pause time between each stroke, $t_{\text{pause}}\in \left[0.2\;\text{s},\;2.0\;\text{s}\right]$ , the starting position of the object (in front of the fin or centered in the tank), and the number of motors in the corners of the tank (one, two, or four). By varying a combination of these parameters, a diverse range of interactions between water surface environments and objects can be created, allowing the emergence of multiple limit cycles. Using the default parameters of the motor, the actuation is performed in step mode such as presented in the parameterized controller plot (Figure 1D) and each servos is actuated according to the selected motor sequence. The actuation time, *t*_move_, is managed by the system to reach the specified amplitude, *A*, while the pause time *t*_pause_ (later *t*_p_) is a parameter of the experiment plan.

### 2.2 Floating objects and CV tracking

**FIGURE 2:**
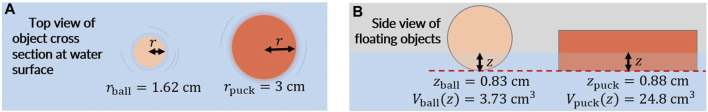
**(A)** Top view of the floating objects’ cross section with the calculated radius at the water surface. **(B)** Side view of the floating objects with the submerged distance and volume indicated.

Since the morphology of the floating object has a significant effect on its trajectory on the fluidic environment surface, several items are tested in this study, as shown in Figure 1B. They comprise of table tennis balls (later “ball”), as well as 3D printed ice-pucks (later “puck”), which are hollow and disk-shaped. The buoyancy Eq. 1 can be rearranged to Eq. 2, where ${\vec{F}}_{\text{b}}$ is the buoyancy force, *ρ*_w_ is the water density, *V*_w_ is the volume of water displaced by the floating object, *g* is the acceleration due to gravity, *m*_o_ is the mass of the floating object, and *V*_o_(*z*) is the volume of the floating object submerged a distance *z* in the water. From geometry, the total volume submerged in the water for the ball and puck is calculated as Eqs 3, 4, where *R* is the radius of the objects. Figure 2A shows the ball and the puck’s cross section at the water surface with the calculated radius *r* indicated. Figure 2B shows the side view of the floating objects with the submerged distance *z* and the submerged volume *V* indicated.

$${\vec{F}}_{\text{b}}=\rho_{\text{w}}V_{\text{w}}g=m_{\text{o}}g$$ (1)

$$m_{\text{o}}=\rho_{\text{w}}V_{\text{o}}\left(z\right)$$ (2)

$$V_{\text{ball}}\;\left(z\right)=\pi \left(R_{\text{ball}}z^{2}-\frac{1}{3}z^{3}\right)$$ (3)

$$V_{\text{puck}}\;\left(z\right)=\pi \left(R_{\text{puck}}^{2}z\right)$$ (4)

**FIGURE 3:**
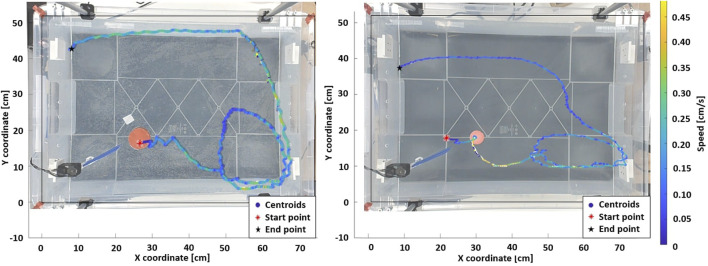
Standard patterns for the puck and the ball (Amplitude = 70°, Pause time = 0.8 s). In both scenario, the fin is actuated for 45 s, then the system is allowed to decay freely in energy for the next 10 s. The loop dimensions in the bottom right corner is related to the object’s geometrical properties.

The trajectory of the floating objects are recorded using a webcam recording at 30 FPS. The camera is mounted perpendicular to the plane of the water surface. To extract the trajectory from the raw videos, each frame is compared to the first frame to obtain a binary difference image. Each of these images is then blurred, and a binary mask is created to detect the largest item of a specified RGB value in each frame, which corresponds to the floating body. The mean location of the object in the two dimensions of the image frames is used to identify the floating item location in each frame. In addition to the Cartesian position of the object, its instantaneous speed is also recorded. To ensure repeatable measurement, a color thresholder algorithm was used to distinguish red markers in the corners of the box from their surroundings, defining them as the reference frame for the tests. Two example curves obtained with the tracking algorithm can be found in Figure 3, respectively for the puck and the ball.

### 2.3 Emergence of limit cycles

Floating objects in a contained environment with actuated waters may follow repeatable trajectories which are similar to limit cycles (Grotmaack and Meylan, 2006; Huang et al., 2011). The experimental setup inherently involves wall effects, but our deliberate choice is to operate within these confines. This allows gaining insight into the conditions under which limit cycles can naturally arise within this specific system environment. In Figure 3, the representative emergent limit cycles for a floating puck and a table tennis ball, actuated by a single fin, are shown. The puck (left) accelerates when approaching a wall due to the waves bouncing off the panel and adding to each other, which increases the object’s speed. In contrast, the ball (right) dissipates its energy over a longer and elliptic clockwise loop compared to the circular trajectory of the puck. This is due to the mass difference between floating items, as the ball is not able to store much potential energy. In addition, from the first fin stroke, the energy transmitted to the ball is not sufficient to make it move far enough from the fin before the second stroke. As a result, the ball receives a second energy boost that significantly increases its speed. The latter effect was consistently observed for this setup configuration.

We observe that emergent limit cycles are influenced by the shape, area, and mass of the object as these factors directly affect the contact surface with the transmission medium (water) and the amount of energy delivered by the motor and fin setup. In addition, we observe that some patterns of behaviour are repeatable and show high controllability, whereas for others this is not the case.

### 2.4 Energy quantification with a single stroke

**FIGURE 4:**
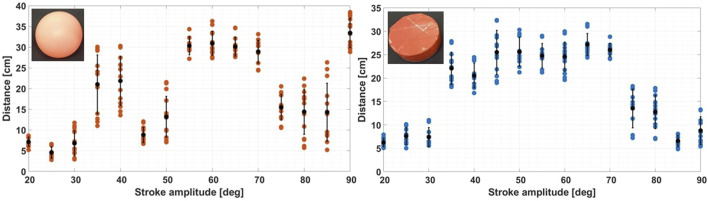
Maximum free displacement after a single back and forth fin stroke for the ball and the puck. The ball reacts periodically to stroke amplitudes, while the puck behave like a band-pass filter. For this plot, each sequence is performed 15 times with clear outliers (due to tracking errors) removed.

Energy is a crucial parameter in characterizing this system, and in order to quantify it, object displacement is recorded when a fin is actuated with a single back-and-forth stroke. Each test is performed 15 times with clear outliers (due to tracking errors) removed. Figure 4 shows the relationship between the amplitude of the fin stroke and the distance traveled by floating items. Black points and error bars represent the mean value and standard deviation for each stroke amplitude.

For both bodies, energy is injected in the system by the motor to make the object travel significantly (a minimum of 35 degrees of fin amplitude). On the left plot (for the ball), the ball reacts periodically for different stroke amplitudes and travels farthest distances around specified amplitude ranges (35–40° and 55–70°) with low deviation at range limits. From 80 degrees of amplitude, ball movements on the water surface become chaotic, which leads to poorly repeatable trajectories. On the right plot, the puck reacts like a band-pass filter, with an ideal energy transfer range from 35 to 70 degrees of fin amplitude. For amplitudes above 70°, both objects bounce on waves and spin on their yaw axis, decreasing the total amount of total energy available for travelling. Single-stroke measurements reveal that an object’s stored energy in a closed water environment is primarily influenced by its shape, size, and mass. Notably, the puck, being approximately six times heavier and larger in submerged volume than the ball, exhibits stability and a wide energy transfer range. Varying the stroke angle of the fin can significantly vary the energy and hence motion of the floating object. Due to this study and analysis of the stroke amplitude, we parameterize the motion of the fin by stroke angle and pause time, i.e., the amount of time this energy is given to dissapate before injecting additional energy.

### 2.5 Similarity identification between trajectories

**FIGURE 5:**
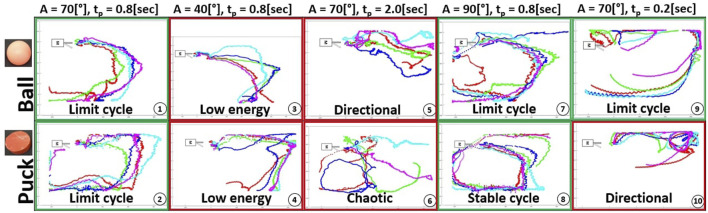
Experiment plan results for a single motor setup - Start position in front of the fin. From left to right: Standard parameters, lower amplitude, longer pause time, higher amplitude and longer pause time. Each sequence is repeated 5 times with similar parameters. Green and red frames around each sub-figure are indicative markers of the presence or absence of limit cycles.

To evaluate the similarity or repeatability of repeated trajectories we choose to use dynamic time warping (DTW) to adjust the trajectories and then generate a metric for error. This enables us to test the correlation between each patterns of floating item with analog parameters but which may vary in speed. Results from the single motor setup experiment plan were used (see Figure 5). To make this analysis each trajectory was compared to a representative curve within each amplitude and pause time configuration. As computer vision tended to generate outlier centroids at the end of each take, only the first half of data-points are considered for the DTW study.

## 3 Results

### 3.1 Experimental plan for limit cycle characterization

An experimental plan was developed to evaluate the impact of key parameters on the setup shown in Figure 1A. All sequences were performed with a fin actuation time of 45 s, followed by an additional 10 s without actuation to allow a free energy decay into the system. For single stroke measurements, 20 s of tracking time were adequate to enable the floating object to dissipate its energy.

The experimental plan is threefold. First, a single motor setup is used where the variable parameters are the fin amplitude and the pause time. This is used to characterize the system’s physical properties (dimensions and wall adhesion). Figure 3 shown earlier in Section 2.3 is part of the dataset collected for this portion. Second, a dual motor setup in a diagonal configuration is used, where the variable parameters are unchanged from the first. The object is released in the middle of the tank, to characterize the different floating items tested, in terms of geometry and weights. Finally, we demonstrate an open-loop control of the floating objects with modifications to the motor sequence.

### 3.2 Limit cycles in a single motor environment

Figure 5 shows the trajectories of the floating objects when the environment is actuated using a single motor. Each sequence with fixed parameters was performed 5 times and the objects are released in front of the fin.

Subplots 1 and 2 demonstrate the presence of limit cycles when using the standard parameters, as similar initial positions lead to comparable patterns. With a lower fin amplitude (subplots 3–4), the total energy in the system declines, and the ball is subject to bouncing wavelets, travelling shorter distances. However, the puck retains enough energy to reach the opposite wall. By increasing the amplitude (subplots 7–8), the floating object obtains enough energy to complete a full circuit of the closed system. Due to its greater contact surface and weight, the puck follows constant trajectories with minimal deviations within each loop. On the contrary, the ball is influenced by its initial placement on water, and the first strokes have a significant impact on the path.

With a longer pause time (subplots 5–6), the object can move freely. As a result, the ball advances easily, while the puck trajectory is influenced by even slight changes in the starting position. Finally, with a shorter pause time (subplots 9–10), the object receives energy in small and regular doses, resulting in smoother patterns. The system energy is higher than under standard conditions, as the ball follows the walls for the first half of its trajectory, while the puck needs at least one clockwise loop in a corner to lose its energy.

The system displays emergent limit cycles for various amplitudes and pause times. The floating object’s geometry and weight play a crucial role in determining the path, as two different objects follow contrasting paths. Additionally, multiple sources of noise including wavelets, surface tension, and bouncing waves on panels were recognized as having a significant impact on limit cycles.

### 3.3 Trajectory similarities for objects floating in a single motor environment

To test the correlation between each patterns of floating objects with analog parameters but which may vary in speed, results from the single motor setup experiment plan were used (see Figure 5) in a dynamic time warping (DTW) algorithm. Each pattern was compared to the average curve within each amplitude and pause time configuration. As computer vision tended to generate outliers towards the end of the trajectories, only the first half of trajectory is considered for the DTW study.

**FIGURE 6:**
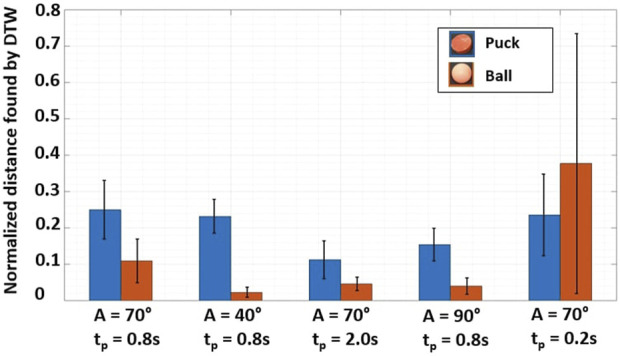
Normalized distance found by Dynamical Time Warping, between the trajectories for limit cycles with a single motor (Figure 5) and a representative trajectory. Blue bars are related to the Puck, while orange bars are related to the Ball. Excluding the final results *A* = 70° and *t*_*p*_ = 0.2 s, the normalized energy is consistently lower for the ball as compared to the puck. DTW is therefore a valid way to sort objects based on their energy.

Figure 6 presents the results for this configuration. The error bars represent the standard deviation associated to the 5 tests of each sequence. Based on the results obtained from dynamic time warping, several observations can be made. When excluding the final results which correspond to a shorter pause time configuration (*A* = 70° and *t*_*p*_ = 0.2 s), it is evident that the normalized energy required to fit each 5-pack curve from Figure 5 to the representative curve is consistently lower for the ball as compared to the puck. The error bars present in the results reflect inconsistencies rising from the computer vision process, leading to continuity errors within the computation of the mean path used as reference.

DTW, applied to both ball and puck curves, highlights disparities in energy requirements. This observation suggests that trajectory tracking and the observed variation can potentially convey insights about an object’s type and mass (and submerged volume). In earlier energy quantification experiments (Figure 4), the puck which is six times heavier than the ball displayed greater stability for single-strokes across a wider range of actuator inputs. However, the ball demonstrates a higher propensity for limit-cycle-like behavior due to its inherent instability within a repetitively actuated environment.

In addition, it can be seen that the error bars and DTW metrics are the lowest for the middle 3 ball configurations. Even through the associated trajectories were labelled respectively as *Low energy, Directional and Limit cycle*, the algorithm has indicated the movement has a high repeatability.

### 3.4 Limit cycles in a dual motor environment

**FIGURE 7:**
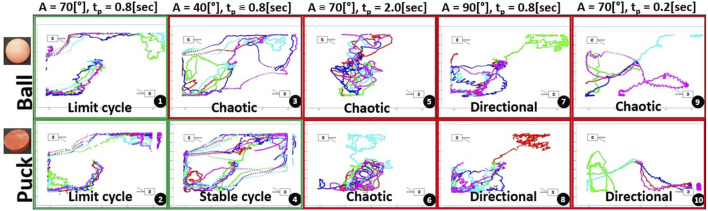
Experiment results for a dual motor setup—Start position in the middle of the system. From left to right: Standard parameters, lower amplitude, longer pause time, higher amplitude and longer pause time. Each sequence is repeated 5 times with similar parameters. Green and red frame are indicative markers of the presence or absence of limit cycles.

Figure 7 shows the trajectories of the floating objects, where the water is actuated by two fins. Each sequence with fixed parameters was performed 5 times and the objects were released from starting in the middle of the tank. As depicted in subplots 1–2, the trajectories are highly similar when using standard amplitude and pause time. The object departs from the unstable middle position of the storage box, bouncing on wavelets as the trajectory is tangent to the waves generated by the fin strokes. Both objects then follow the clockwise current towards the top left motor, experiencing re-acceleration at the top left fin within the 45 s of the experiment. This process can be highly repetitious.

A smaller amplitude (subplot 4) results in distinct emergent limit cycles for the puck only (this behavior was experimentally verified over 10 loops). The ball exhibits similar behavior, but the pattern is inconsistent due to the additional wavelets created by the second motor while the ball is in the middle position. With maximal amplitude (subplots 7–8) or a small pause time (subplots 9–10), the floating objects receive an excess of energy and wavelets from the two motors, leading them to a dead end in one of the corners opposite a motor rather than a limit cycle path. Finally, with a pause time of 2 s, both objects react chaotically to the free time between impulses within the system, as shown in subplots 5 and 6.

For the dual motor configuration, we can make the following statements. First, emergent limit cycles taken by the floating objects tend to oscillate due to increased energy at the water surface. Second, it would be possible to draw a potential force field map of the system by analyzing the behavior of multiple object trajectories within the system and their frequency of passage at each point. From present observations, we can identify special points, either unstable (the middle of the system, in front of each motor) or stable (the two free corners and their surrounding walls). Third, the shapes of waves generated by the back and forth movements of the fins have a significant impact on the limit cycles during the re-acceleration of the objects. Different wave shapes, such as punctual ones, would lead to different patterns within the closed system.

### 3.5 Open-loop trajectory control

To demonstrate open-loop control of floating objects, we determine the motor sequence required for non-contact manipulation of an object along a specified trajectory. Optimizing this control requires determining the motor sequence, amplitude, and pause time of each motor and fin setup. A valid motor sequence is a subtle mix of motor order, with sweeped amplitude and pause time. To allow a direction change of the floating item, at least 2 motors should be actuated one after another multiple times with varying amplitudes, to avoid discontinuities (i.e., object ends up in a corner). Additionally, it is wiser to actuate the fins with small amplitude and pause time (around *A* = 40° and *t*_*p*_ = 0.3 s), as it allows more controllable trajectories and avoids any overflows of energy into the system, which would make the system chaotic and unpredictable. The selected amplitude comes from the conclusions of Figure 4, as amplitudes below *A* = 40° are not sufficient to give directional energy impulses to the floating object. Based upon the experimental data and heuristic evaluation, a sequence of control inputs for the two inputs can be chosen to meet a desired pattern.

The starting position of the object is key to avoid a trajectory that collides with a wall. We consider the floating object to be under control as long as it stays within the central rectangle drawn by the four motor positions, which is relatively small compared to the total area of the storage box selected.

**FIGURE 8:**
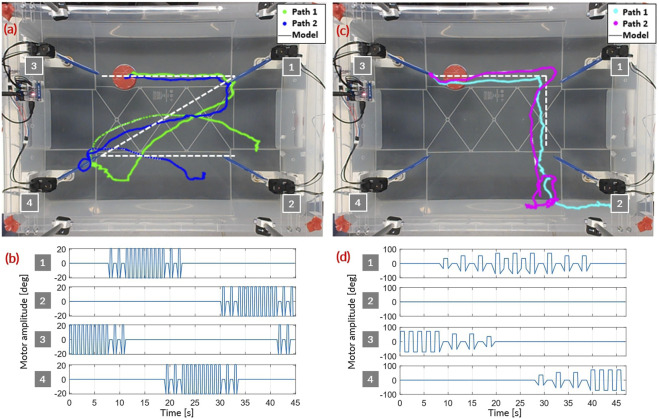
Paths in open loop following Z-shaped **(A)** and inverted L-shaped **(C)** pattern with the corresponding motor sequence **(B)** and **(D)**, respectively. Motor sequences are a subtle mix of motor order, with motor sweep amplitude and pause time. The two open-loop patterns are fully repeatable between the top two motors. The trajectory is then heavily influenced by how the object catches the waves coming from the top right fin (motor #1).

Figure 8 presents two patterns (Z-shape and L-shape) obtained with open loop shape generation with the corresponding motor sequences also shown, which are obtained from heuristics from the experimental results in Figures 4, 5, 7. These were chosen as they reflect some of the motion ‘primitives’ seen in the characterization data. Other sequences were tested to generate patterns, but these results were discarded due to their poor repeatability caused by the multiplication of wavelets over time, as well as the need for adaptive offset tuning (i.e., asymmetrical fin amplitude when the object is approaching a motor position).

As shown in Figure 8, both open-loop patterns show high repeatability for the first section of the trajectory (the horizontal motion). The trajectory is then heavily influenced by how the object catches the waves coming from the top right fin. For the Z-shaped pattern, with the most precise catch (blue path), the puck finishes exactly at the bottom left fin and receives a direct re-acceleration in the correct direction. With a slight stroke delay (green path), the puck’s behavior is influenced by water currents and the re-acceleration in the bottom corner is unstable, leading to an incorrect end trajectory. For the L-shaped pattern, similar behaviours can be highlighted: a direct wave catch (cyan path) allows the floating object to make a clean 90° turn, while with a short delay (purple path) the floating object tends to follow a loose trajectory and wastes additional energy along the way, bouncing on small wavelets generated by the two right motors.

As seen in the single and dual motor setups, the initial position is important in determining the overall path of the object, and the present open-loop control sequences illustrate this evidence once more.

## 4 Discussion

The present study identifies all the main interactions between water and objects within a contained environment. The behavior of floating objects in three different configurations (single and dual motors, open-loop control) could be predicted based on observations and initial parameters.

Based on the single fin system, it was determined that the amplitude of fin movements must be maximized to create an emergent limit cycle, but only up to a certain energy peak (around 70^*o*^ in the present setup, as presented in Figure 4). Beyond this amplitude, the system exhibits chaotic behavior due to excessive energy and wavelets. Additionally, it was observed that the more motors present in the system, the more chaotic it tends to become because equilibrium points become harder to maintain due to stronger currents.

**FIGURE 9:**
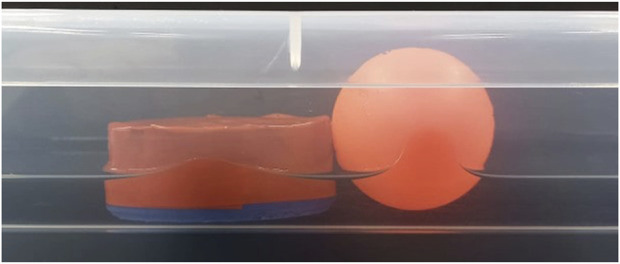
Surface tension of objects in contact with the walls. When the object becomes wet and comes into contact with a wall, surface tension tends to cause it to adhere to the panel inversely proportional to the body’s weight. The ball is therefore more prone to this phenomenon.

Based on our current observations, the most substantial source of noise affecting patterns appears to be surface tension, particularly concerning the adhesion of objects to the panel (Figure 9). Notably, this effect is most pronounced with the ball. Additional sources of noise include underwater currents, surface wavelets, and panel-induced bouncing waves, all contingent on the floating object’s shape. While we acknowledge the intricate nature of these interactions, our research approach prioritizes offering a high-level representation of system behavior and interactions, as illustrated through the concept of limit cycles. Our primary focus is on comprehending the underlying conditions and mechanisms driving these cycles, where the emergence of limit cycles are defined by each particular system.

To continue researching using the present system, the next step would be to implement a closed-loop control algorithm with adaptive fin offsets. All patterns obtained through this study were rounded at the corners due to the movement of the fins, which oscillate around a determined equilibrium position. By using smaller and constant fin strokes (around the first energy peak of 40^*o*^ for the ball) with a dynamically changing fin middle position, it would be possible to sharpen patterns and more accurately direct floating objects. This control technique, which is fully implementable in a food processing plant, belongs on the list of significant improvements that can be made on industrial food lines.

One potential enhancement to consider is to modify the rectangular fin shape that moves back and forth through the water by replacing it with a conical fin that moves up and down through the water’s surface. This modification would alter the wave pattern from a series of spherical fronts to a point source, resulting in a uniform distribution of energy within each stroke and greater ease in directing floating objects. The energy transmitted by point wave sources is also easier to quantify, and the current method of indirect energy quantification by measuring the farthest distance traveled by the object would become unnecessary.

Ultimately, our current understanding of non-contact manipulation and energy transfer through water interactions, as obtained from the present experimental plan, is still limited. There is much more for us to learn from the behaviors of marine species and birds in this regard. By continuing to study these natural examples, we can also explore novel ways in which non-contact manipulation can be applied to robotics, for example, of self-assembly or manipulation (Obayashi et al., 2023).

## Data Availability

The raw data supporting the conclusion of this article will be made available by the authors, without undue reservation.

## Footnotes

1 The Great Bubble Barrier, https://thegreatbubblebarrier.com/
